# Magnetic nanobiosorbent (MG‐Chi/Fe_3_O_4_) for dispersive solid‐phase extraction of Cu(II), Pb(II), and Cd(II) followed by flame atomic absorption spectrometry determination

**DOI:** 10.1049/nbt2.12025

**Published:** 2021-02-19

**Authors:** Narges Salehi, Ali Moghimi, Hamidreza Shahbazi

**Affiliations:** ^1^ Department of Chemistry Varamine (Pishva) Branch Islamic Azad University Varamin Iran

## Abstract

Trace amounts of Cu (II), Pb (II), and Cd (II) in a wastewater sample were preconcentrated with a novel cross‐linked magnetic chitosan modified with a new synthesised methionine‐glutaraldehyde Schiff's base (MG‐Chi/Fe_3_O_4_) as a dispersive solid‐phase extraction (DSPE) adsorbent. The adsorbed metal ions were then eluted with a specific volume of suitable solution and determined by flame atomic absorption spectrometry (FAAS). Various parameters affecting the extraction efficiency of the metal ions were investigated and optimised, including pH, amount of adsorbent, extraction time, type and volume rate of eluent, elution time, sample volume, and effect of interfering ions. The adsorption kinetics are more consistent with the pseudo‐second order model. The results were statistically interpreted and the analytical performance of the proposed method was found to have preconcentration factors of 55, 60, and 50 μg L^−1^ for Cu(II), Pb(II), and Cd(II), respectively, limits of detection were 0.22, 0.24, and 0.10 μg L^−1^ for Cu(II), Pb(II), and Cd(II), respectively, with a relative standard deviation (1.5%‐2.8 %), and the liner range was 5–1000 for Cu(II) and Pb(II) and 2.5–1000 for Cd(II). It was concluded that this method was suitable for successful simultaneous determination of Cu(II), Pb(II), and Cd(II) in industrial wastewater samples.

## 1 INTRODUCTION

Because of industrial and manufacturing developments, heavy metals enter environmental water and subsequently, due to their non‐biodegradability, they enter the human body through the food chain and, if they exceed the normal range, may potentially cause toxicity and endanger human health [1]. The low concentrations of these metal ions and the presence of a complex matrix make it difficult to measure, despite having possession of modern, sensitive and analytical instruments. To solve this problem, separation and preconcentration are needed to eliminate the effects of the matrix and improve the detection limit. Various separation techniques including coprecipitation, ion exchange, and most of all, solid‐phase extraction (SPE) methods, for example magnetic SPE for measurement of heavy metal ions, have been used in environmental waters [2]. In SPE, instead of two dispersive phases such as liquid–Liquid extraction (LLE), there is a split between the liquid (sample matrix) and solid phases (adsorbent). This conventional filtration technique provides the concentration of the sample of the solution by adsorption on an adsorbent [3]. This technique has been used successfully recently in sample preparation [4] and the separation of metal ions, especially in environmental waters [5].

Dispersive solid‐phase extraction (DSPE) was first recommended by Anastassiades et al. as an alternative to conventional SPE [6]. This method accelerates the reaction between analyte and adsorbent particles, shortens the sample preparation time, and has high extraction efficiency compared to classical SPE. Adsorbents play an important role in improving the performance of an analytical method. Therefore, the new adsorbents have attracted the attention of researchers [7, 8]. The most important feature of magnetic solid phase extraction (MSPE) is that magnetic nanoparticles (MNPs) are adsorbed by the desired components and easily separated from sample solutions by a magnet without centrifugation or sample purification, making sampling and collection easier and faster [9].

Nano‐sized magnetic adsorbents, due to their high surface area and high adsorption capacity, are of great importance for use in MSPE techniques. Therefore, small amounts of these adsorbents at short equilibrium time are sufficient to separate the analyte from a large amount of sample [10]. Conventional SPE adsorbents have limitations such as low selectivity, low adsorption capacity, and low stability during extraction. The selectivity of the SPE method can be improved with chelating resins [11], carbon nanotubes [12], polymeric adsorbents [13], and imprinted polymers [14, 15]. Recently, due to biosorbent biocompatibility and low cost, the adsorption of heavy metals in industrial wastewaters by biosorbents has received growing attention [16, 17]. Chitosan is one of the most environmentally friendly and biodegradable polymers, with strong adsorption properties and broad functional potential [18, 19]. The functional groups on chitosan such as amine and hydroxyl may cause an electrostatic reaction for metal ions [20, 21]. However, chitosan has been cross‐linked by chemical reagents such as tripolyphosphate, gentioine, epichlorohydrine, glutaraldehyde, oxidised beta‐cyclodextrin, and ethylene glycol diglycidyl ether due to its low acid stability and mechanical properties. [22]. Cross‐linked chitosan has a binding site (e.g. __COOH, __NH_2_, __CN, and __S^__^) for soft cations [Cd(II)] and intermediate behaviour cations [Cu(II) and Pb(II)] [3, 23].

The purpose of the present study is the synthesis of a new magnetic nanobiosorbent (modified magnetic cross‐linked chitosan by methionine‐glutaraldehyde) to shorten the sample preparation time compared with classic SPE and enable easier separation and preconcentration of Cu (II), Pb (II), and Cd (II) in DSPE before determination by flame atomic absorption spectroscopy (FAAS). By modifying chitosan and forming functional groups (e.g. __COOH, __NH_2_, __CN, and especially __S^__^), soft metal ions [Cd(II)] and intermediate behaviour cations [Cu(II) and Pb(II)] are well adsorbed on the sorbent [22]. After characterisation of the new magnetic nanobiosorbent and influencing parameters on adsorption, this method was successfully applied for the preconcentration and determination of Cu(II), Pb(II), and Cd(II) in industrial wastewater samples.

## 2 EXPERIMENT

### 2.1 Reagents and materials

Samples of [Pb(NO₃)₂], [Cu(NO₃)₂.3H₂O], and [Cd(NO_3_)_2_] (purity > 99.5%), were acquired from Merck (Darmstadt, Germany). Individual stock solutions of Cu (II), Cd (II), and Pb (II) were prepared by direct dissolution of the appropriate amount of salt in 1% HNO_3_. By stepwise dilution of the stock solutions, a mixed working solution was prepared. Chitosan was purchased from Sigma‐Aldrich (Darmstadt, Germany), with a deacetylation rate > 90%, nitrate salt of Cu (II), Cd (II), and Pb (II), magnetite particle (Fe_3_O_4_) with particle size less than 20 nm, glutaraldehyde 25%, piperidine, ethylene diamine tetra acetic acid (EDTA), and ethanol absolute (all with purity greater than 99.5%) and methionine (with purity greater than 99%) were prepared from Merck (Darmstadt, Germany).The pH of the solution was adjusted by the addition 2 ml of acetate buffer (acetic acid/sodium acetate) 1.0 mol L^−1^ with pH = 5.5.

### 2.2 Apparatus

The Fourier transform infrared (FTIR) spectra at every stage in the synthesis of magnetic nanobiosorbent were recorded using an FTIR spectrometer in the range 4000–400 cm^−1^ using the KBr pellet technique (Thermo, AVATAR, Massachusetts, United States). Magnetic separation was fulfilled using a supermagnet with 1.2 T magnetic field (N35 model from Tehran Magnet, Tehran, Iran). For determination of the metal ions, a flame atomic absorption spectrometer (FAAS) (Varian Spectra AA 200, Australia) was applied. The size and morphology of the magnetic nanobiosorbent were characterised by scanning electron microscope (SEM, PHILIPS, CM120, Amsterdam, Netherlands). For characterisation of the crystallinity of the magnetic nanobiosorbent, X‐ray diffraction (XRD) (from Philips, PW1730, Amsterdam, Netherlands) was applied.

### 2.3 Synthesis of the magnetic nanobioadsorbent beads (MG‐Chi/Fe_3_O_4_)

In a round flask, 1.0 ml glacial acetic acid and 2.0 ml of glutaraldhyde were added to 30 ml ethanol absolute and then heated to 80°C. Also, 1.21 g methionine was suspended in 10 ml ethanol absolute and heated to 120°C and then added to the previous mixture. The final mixture was refluxed at 120°C for 24 h. Then, the created precipitate was eluted by 50 ml hot ethanol and dried in a desiccator for 48 h.

One g of nanoparticles was suspended in the solution in which the 3.0 g of chitosan was dissolved in 100 ml aqueous acetic acid solution (1.0%, v/v) and mixed for 4.0 h in which time it became homogeneous and was dropwise added into ethanolic solution of sodium hydroxide (1.0 mol L^−1^) by a 1 mm in diameter syringe needle. Gelatinous beads of magnetic chitosan were immediately precipitated. The precipitated product was eluted by distilled water followed by ethanol.

**FIGURE 1:**
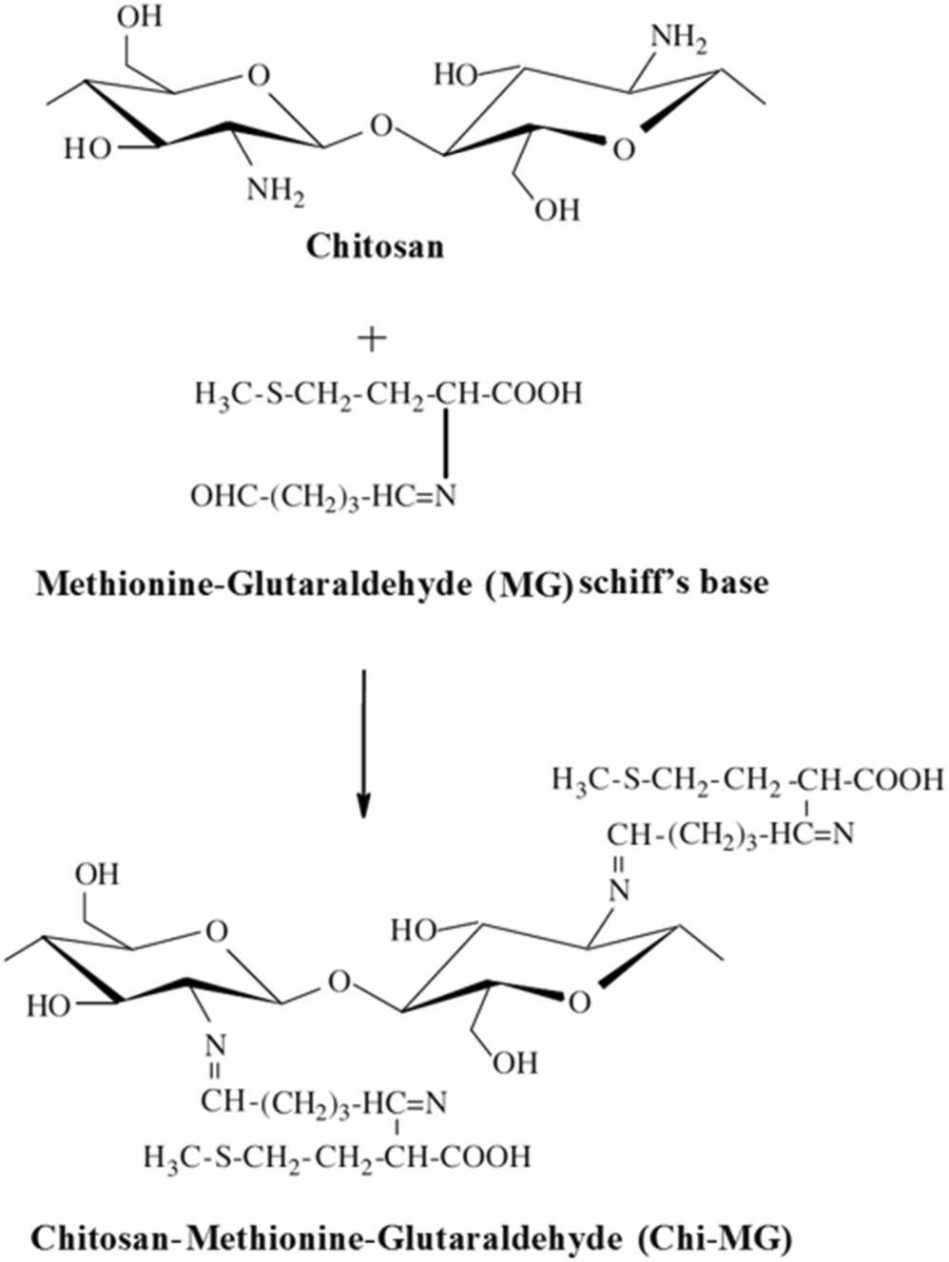
Cross‐linked Chi‐MG magnetic beads were synthesised via Schiff's base formation between the amino group in chitosan and the active carbonyl group of methionine

One g of MG, 2.0 ml glutaraldehyde, 100 µL piperidine, and 3 g of the magnetic beads were dissolved in 100 ml ethanol. The final solution was refluxed at 120°C for 48 h, and the product was eluted by ethanol elution. The final product was dried at 40°C and powdered [23, 24]. Figure 1 shows the form of the desired adsorbent.

### 2.4 Preconcentration procedure experiments

**FIGURE 2:**
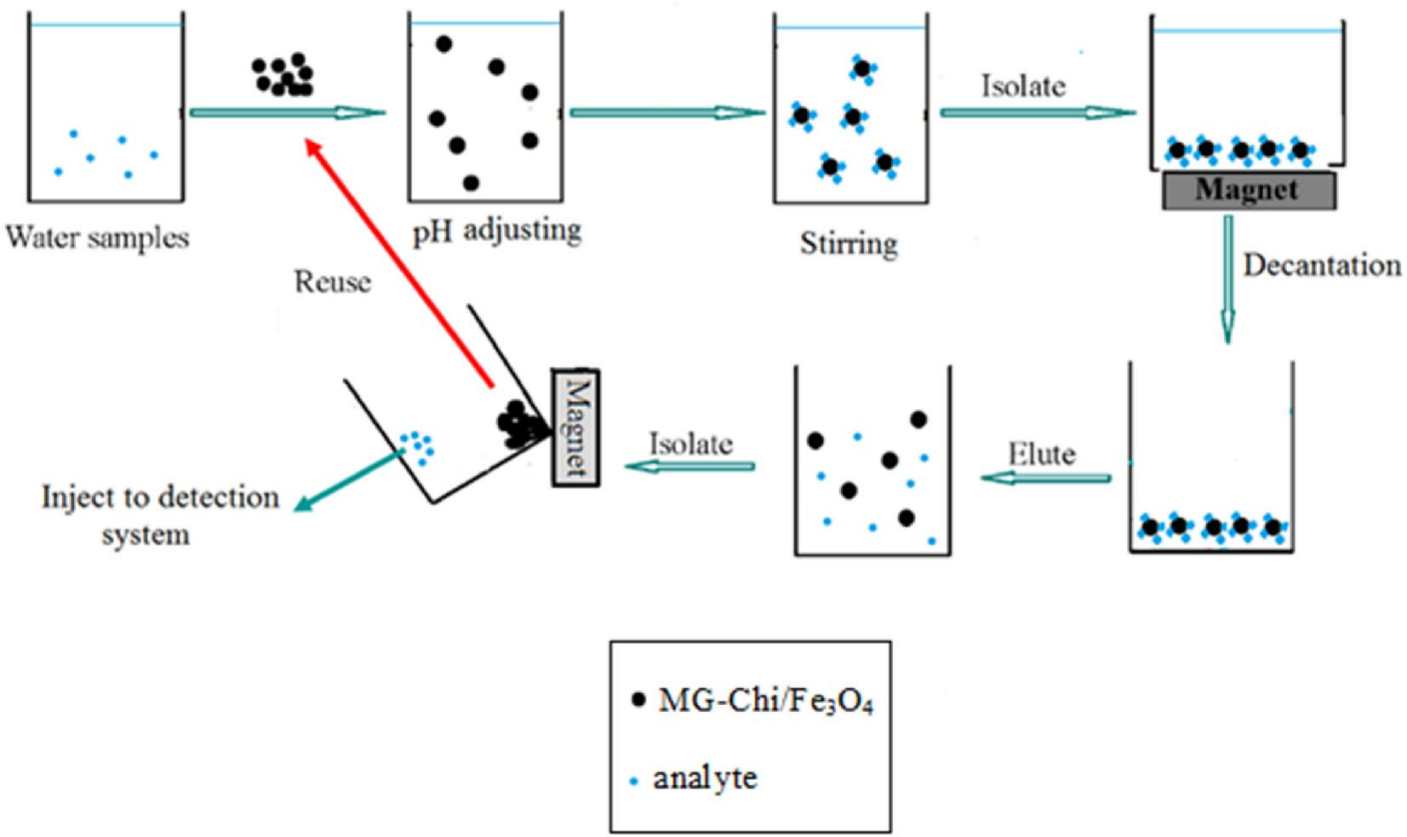
Preconcentration procedure experiments

In each experiment, the pH of 100 ml of the mix metal ion solution in a 150 ml flask was adjusted to 5.5 by the addition of 2 ml of the buffer solution (acetic acid/acetate 1.0 mol L^–1^) and then 20 mg of the magnetic nanobiosorbent was added, the solution was stirred for 5 min by mechanical stirring. The mixture was then separated by an external magnetic field (magnet) from the magnetic nanobiosorbent and was eluted for 2 min by 2 ml of EDTA (0.1 mol L^−1^)., The metal ions were determined by atomic absorption spectrometry after desorption (Figure 2). These samples were tested three times.

## 3 RESULTS AND DISCUSSION

### 3.1 Characterisation

#### 3.1.1 IR spectra analysis

**FIGURE 3:**
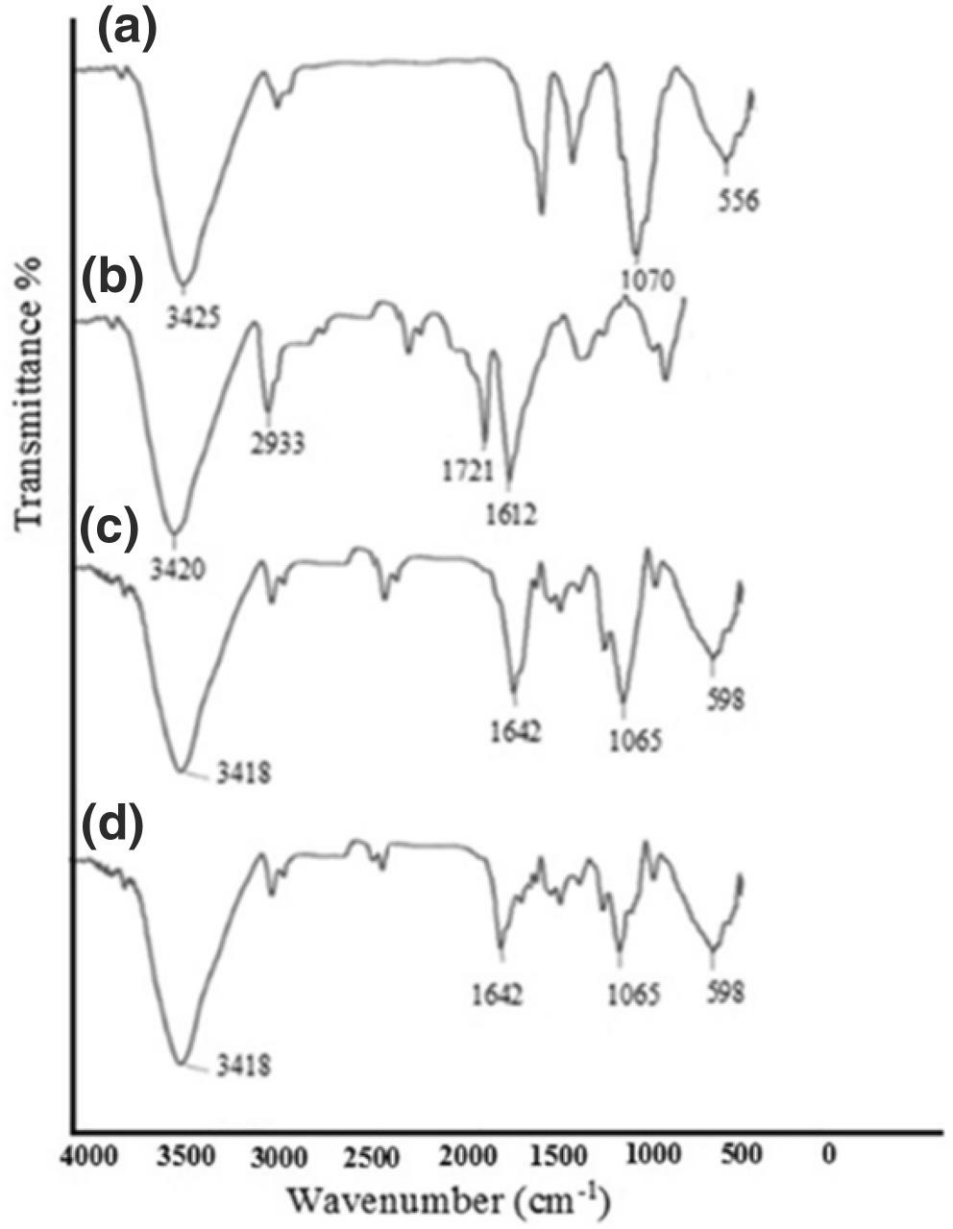
FT‐IR spectra of (a) Chi/Fe_3_O_4_, (b) MG, (c) MG‐Chi/Fe_3_O_4_ and (d) MG‐Chi/Fe_3_O_4_‐Cu

In Figure 3 the FTIR spectra of (a) MG, (b) Chi/Fe_3_O_4_, (c) MG‐Chi/Fe_3_O_4_, and (d) MG‐Chi/Fe_3_O_4_‐Cu are shown. In the Chi/Fe_3_O_4_ spectrum, the stretching vibrations at 3425 cm^−1^, 1070 cm^−1^, and 556 cm^−1^ can be related to the stretching vibration of hydroxyl, __C^__^O, and Fe^__^O, respectively [25]. In the MG spectrum, peaks in the areas at 2933 cm^−1^, 1612 cm^−1^, and 1721 cm^−1^ can be assigned in relation to aliphatic C^__^H, __CN, and C ^‗^ O vibrations, respectively.

The vibration of __CN after bonding Chi/Fe_3_O_4_ and MG that was not observed in both previous spectra, probably was shown in 1642 cm^−1^ in the Chi‐MG/Fe_3_O_4_ spectrum. The decreased intensity of peaks at 1642 cm^−1^ and 1065 cm^−1^ in the MG‐Chi/Fe_3_O_4_‐Cu spectrum was probably due to conflict of the bonding sites in the uptake of Cu(II) [23, 24].

#### 3.1.2 XRD analysis

**FIGURE 4:**
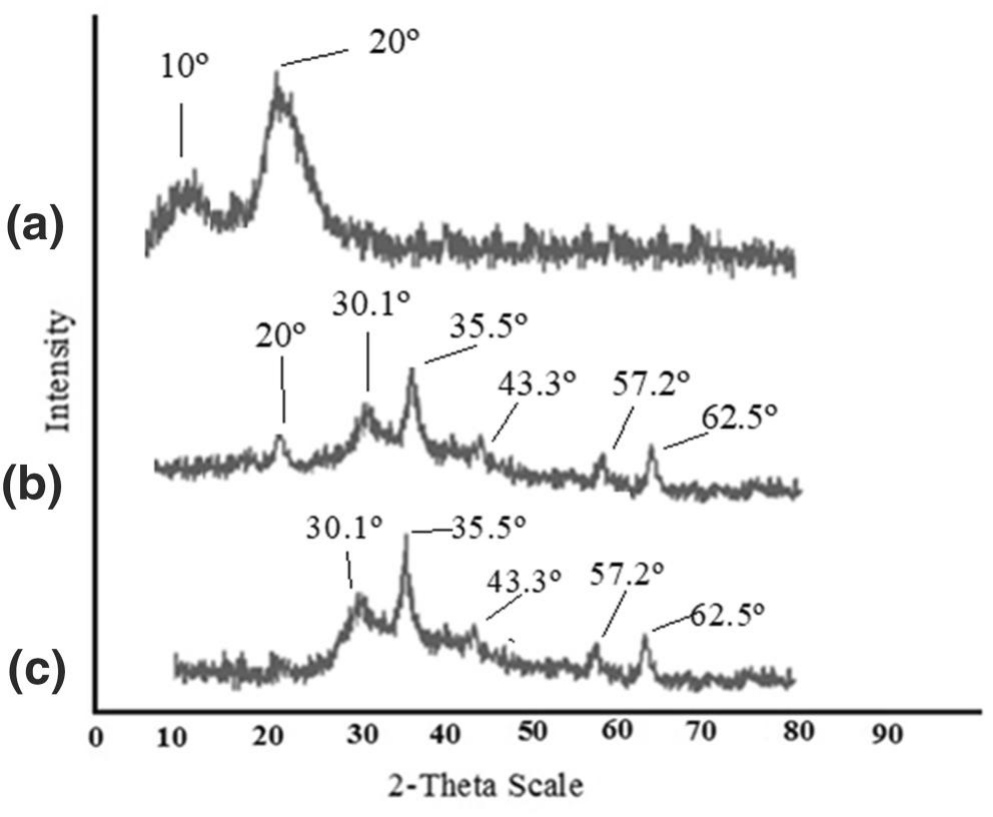
X‐ray diffraction patterns of (a) Chi, (b) MG‐Chi/Fe_3_O_4_, and (c) Fe_3_O_4_

Figure 4 shows the XRD patterns of (a) Chi, (b) MG‐Chi/Fe_3_O_4_, and (c) Fe_3_O_4_. The peaks at 2*θ* = 10º and 20° in the MG‐Chi/Fe_3_O_4_ pattern were weaker than the pattern for chitosan, which has due to a decrease in the crystallinity of the cross‐linked chitosan [26]. Five typical peaks for Fe_3_O_4_ (2*θ* = 30.1°, 35.5°, 43.3°, 57.2°, and 62.5°) were also found in the XRD pattern of the sorbent with lower intensity [23, 24, 25, 26].

#### 3.1.3 SEM images

**FIGURE 5:**
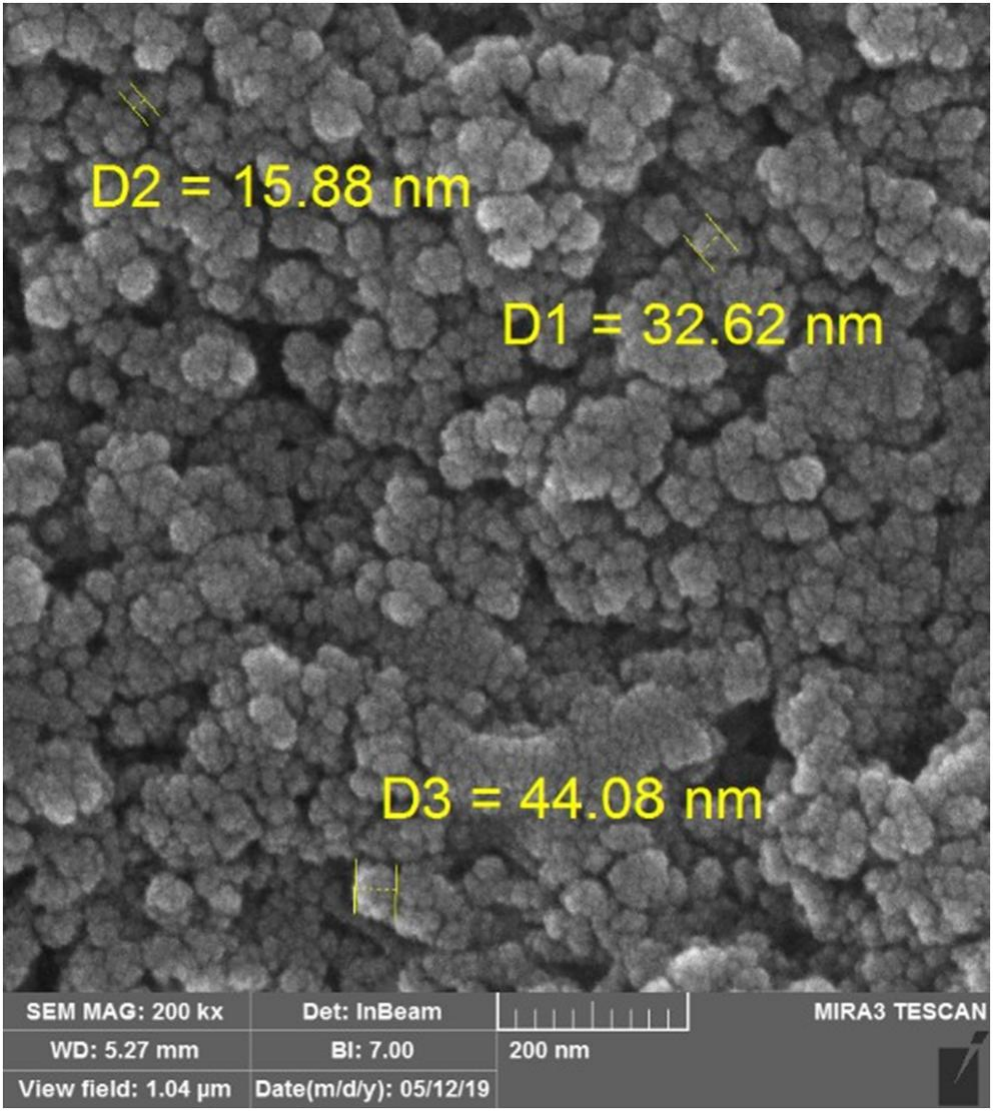
Scanning electron microscope photograph of MG‐Chi/Fe_3_O_4_

A scanning electron microscope was used to determine the size and characteristics of the magnetic bionanosorbent beads as shown in Figure 5.

The particles were observed to be approximately spherical in shape and about 30 nm in diameter.

### 3.2 Optimisation of suggested method variables

#### 3.2.1 Effect of pH

**FIGURE 6:**
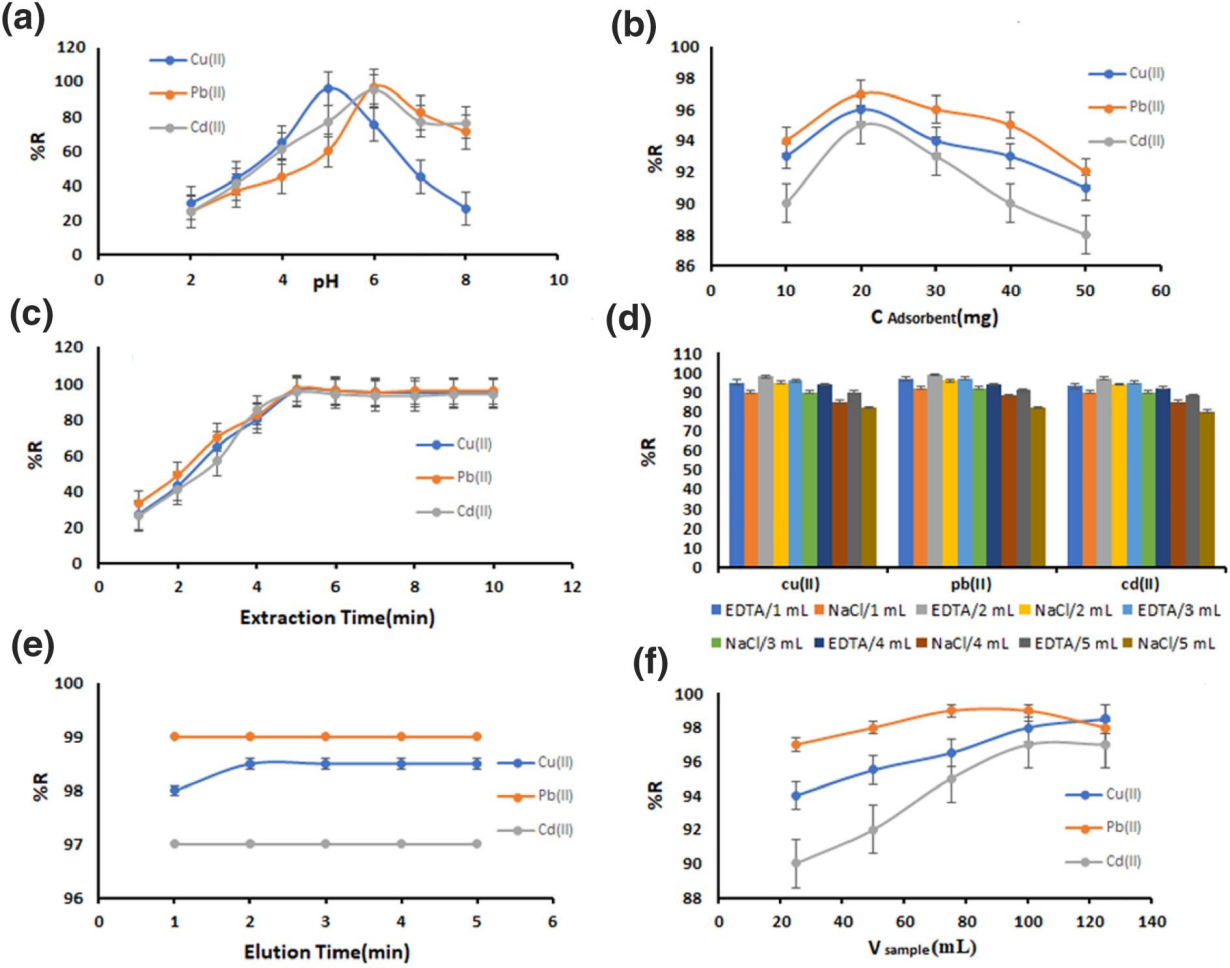
(a) Effect of pH, (b) wt of MG‐Chi/Fe_3_O_4_ (mg), (c) extraction time (min), (d) type and volume of eluent, and elution time (min), and (f) Sample volume (ml). (Experimental parameters: pH in b, c, d, e, f = 5.5, C_e_(M) = 0.5 ppm; wt of MG‐Chi/Fe_3_O_4_ = 20 mg in a, c, d, e, f; Extraction time = 5 min in a, b, d, e, f; type and volume of eluent = 2 ml ethylene diamine tetra acetic acid in a, b, c, e, f; elution time = 2 min in a, b, c, d, f; sample volume = 100 ml in a, b, c, d, e, and temperature = 298 K)

The pH of the sample solution is an important factor for the adsorption process, especially in the basic acid adsorbents and in particular the adsorption capacity by changing the ionisation level of the chitosan derivatives [27]. The dependence of metal adsorption on pH is related to the chemical state of the metals in solution and the ionisation state of the adsorbent functional groups, which affects the availability of binding sites [28]. The pH values chosen for the adsorption experiments were in the range of 0.2–0.8, because at higher pH values the precipitation of metals as hydroxides may occur simultaneously and lead to misinterpretation of the adsorption [26]. In Figure 6(a) it can be observed that the relative recoveries increased by increasing pH. At low pH, the adsorbent was protonated and the intensity of adsorption was decreased. At high pH, the relative recoveries increase with the presence of free lone pairs of electrons of __NH_2_, __CN^__^, and __S^__^, and also easy deprotonation of carboxylic groups. Therefore, coordinating magnetic nanobiosorbent with metal ions was easier [26], and the optimum pH in the metal ion solution was 5.5. Similar results were previously reported by Abou El‐Reash [23].

#### 3.2.2 Effect of the amount of adsorbent

The amount of adsorbent is an important factor in adsorption studies because it determines the adsorbent capacity for the initial concentration of metal ion solutions. In order to optimise the amount of adsorbent used, weights of 10, 20, 30, 40, and 50 mg were added to 100 ml of metal‐containing solution. In Figure 6b, it can be observed that the relative recoveries increased to 20 mg due to the increase in adsorption sites and then the relative recoveries decreased. This result indicates that a large amount of binding site reduces the adsorption [29].

#### 3.2.3 Effect of extraction time

To investigate the effect of extraction time on the efficiency of the method, times of 1–10 min were studied. It was observed that after 5 min the relative recoveries reached almost a constant level (Figure 6(c)). Therefore, 5 min was finally chosen as the optimum extraction time for preconcentration and determination of metal ions. It can be pointed out that in the initial phase of adsorption, unfilled surface sites are available and, after equilibrium, the remaining binding sites would be hard filled, probably due to repulsive forces between heavy metal ions on magnetic nanobiosorbent and sample solution [30]. Similar results were reported by Chen et al. [7].

#### 3.2.4 Effect of type and volume rate of eluent

In order to achieve high preconcentration factors, selecting the appropriate solvent with minimum volume is essential. To investigate this parameter, EDTA (0.1 mol L^−1^) and NaCl (1.0 mol L^−1^) in volumes of 1–5 ml were tested as the eluting solution, and the results showed that 2 ml of EDTA (0.1 mol L^−1^) relevant the most relative recoveries (Figure 6d).

Covalent bonds (chemisorption: chelation and ion exchange) and van der Waals force (physisorption) or electrostatic interactions are two basic mechanisms for the adsorption of metal ions by chitosan that may occur simultaneously [31].

If the EDTA solution can desorb metal ions from the adsorbent, it is believed to be desorbed by chelating, as EDTA is known to be a strong chelating agent [32].

#### 3.2.5 Effect of elution time

In order to optimise the elution time, it was changed from 1 to 5 min. In Figure 6(e), it was observed that the change in elution time did not show any significant difference in relative recoveries except for Cu(II), which had the highest relative recovery in 2 min and it was chosen as the optimum time for adsorbent elution. The fast desorption was probably due to the high chelating power of EDTA [32].

#### 3.2.6 Effect of sample volume

The purpose of measuring the sample volume is to determine the enrichment factor and the maximum sample volume at which the adsorption efficiency for metal ions is high [7]. In volumes above 100 ml of sample, relative recoveries for this method were reduced for lead and did not differ significantly for cadmium and copper (Figure 6f). Thus, a volume of 100 ml was selected for subsequent experiments. Lower recoveries for sample volumes greater than 100 ml were probably explained by the limited contact between the metal ions and bonding site [33].

#### 3.2.7 Effect of interfering ions

In wastewater samples there are many cations and anions of varying concentrations that can affect the extraction of metal ions and cause a negative or positive determination error. The effect of interfering ions is very important and sensitive. In this study, copper, lead, and cadmium were extracted from aqueous samples containing 1000 μg L^−1^ in the presence of various cations and anions at specified concentrations, and the interference limits of these ions were determined. An interfering ion is referred to as an ion that causes a change of over 5% in the absorption signal of the analyte [34]. The results of this study are presented in Table 1. The results showed that most of the interfering ions do not have significant interference in the extraction and determination of lead, copper, and cadmium metal ions.

**TABLE 1:** Effect of interfering ions on the determination of Cu(II), Pb(II) and Cd(II)

Interfering ion	C _metal ions_/C _interfering ion_	% Recovery		
		Cu(II)	Pb(II)	Cd(II)
Cr^3+^	100	98	98	96
Cr^5+^	100	97	98	97
Na^+^	50	98	98	97
Ca^2+^	50	96	99	95
Ni^2+^	50	98	97	96
Mn^2+^	50	98	99	96
Nitrite	100	97	99	96
Nitrate	100	97	99	96
Acetate	100	98	99	96

*Note: n* = 3.

### 3.3 Adsorption capacity

To determine the adsorption capacity of 10 mg L^−1^ metal ions, 20 mg of the adsorbent was used under optimum condition. The metal ion concentration were determined by FAAS. The adsorption capacities of the MG‐Chi/Fe_3_O_4_ were found to be 46, 46.5, and 45 mg g^−1^ for Cu(II), Pb(II), and Cd(II), respectively.

### 3.4 Adsorption kinetics

Two simple kinetic models were applied to investigate the kinetic mechanism of the adsorption process. The pseudo‐first‐order model is as shown in Eq. 1 [24]:

(1) $$1/q_{t}=\left(K_{1}/q_{e}t\right)+\left(1/q_{e}\right)$$

where *q*_*e*_ and *q*_*t*_ (mg g^−1^) are the adsorption capacity at equilibrium and time *t*, respectively, and *K*_1_ (min ^−1^) is the rate constant of the pseudo‐first‐order model.

**FIGURE 7:**
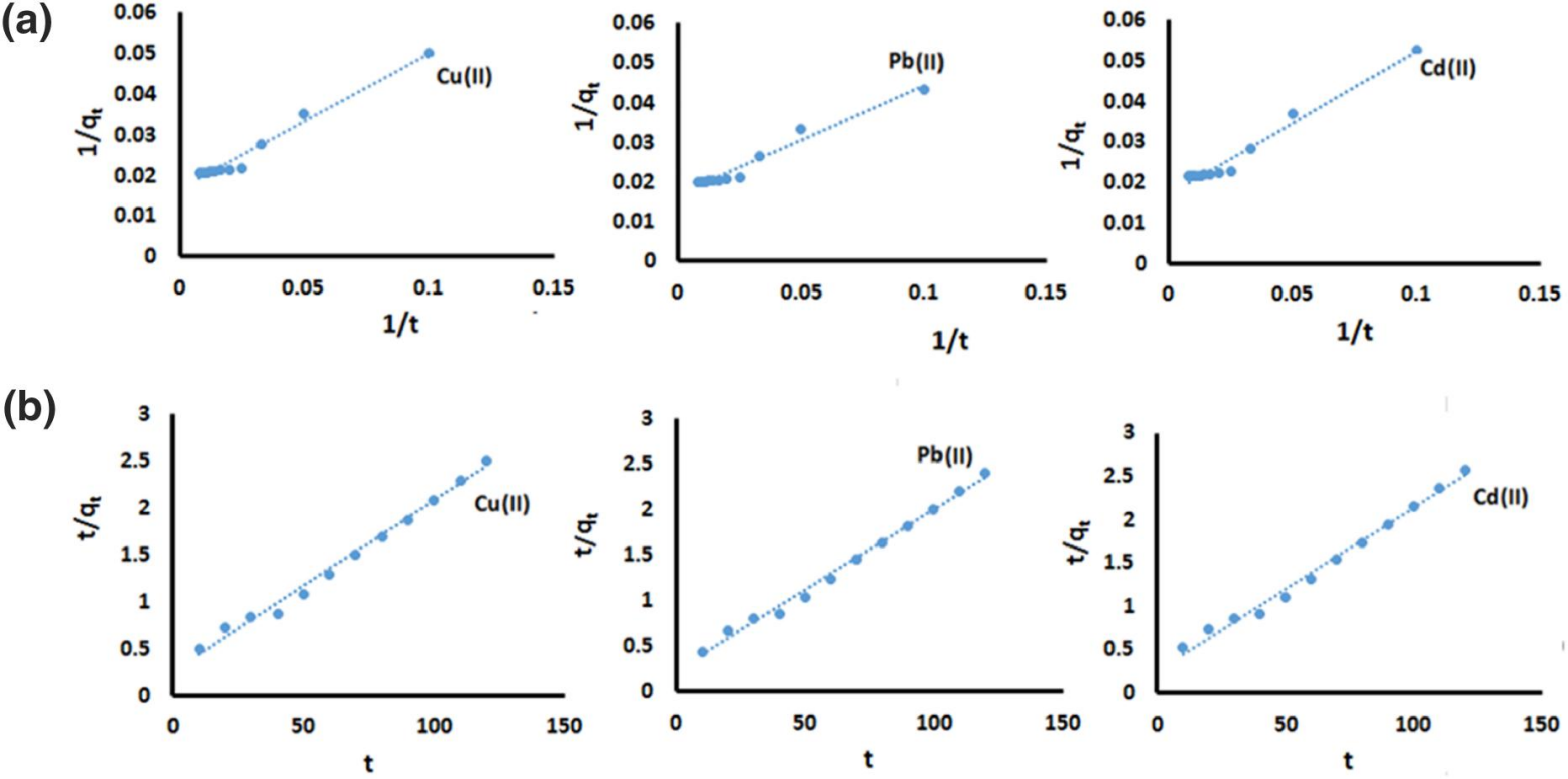
(a) Pseudo‐first‐order kinetic model, (b) pseudo‐second‐order kinetic model for the adsorption of Cu(II), Pb(II), and Cd(II) on MG‐Chi/Fe_3_O_4_

The pseudo‐second‐order [24] is giving in Eq. 2:

(2) $$t/q_{t}=\left(1/K_{2}q_{e}^{2}\right)+\left(t/q_{e}\right)$$

where *K*_2_ (g mg^−1^ min^−1^) is the rate constant of the pseudo‐second‐order model. In Figure 7(a, b), plotted graphs of 1/*q*_*t*_ versus 1/*t* (min^−1^) and *t*/*q*_*t*_ versus 1/*t* (min^−1^) for Cu(II), Pb(II), and Cd(II) are shown. The parameters of the two models are shown in Table 2. The results shown are fitted with the pseudo‐second‐order kinetic model. The absence of mass transfer in the solution and chemical adsorption as a rate‐limiting step are pseudo‐second‐order model assumptions. Due to the amount of *K*_2_ being very small [1.31 × 10^−3^, 1.38 × 10^−3^, and 1.32 × 10^−3^ for Cu(II), Pb(II), and Cd(II), respectively], it can be seen that the absorption equilibrium can occur in a short time [17].

**TABLE 2:** Kinetic model parameters for the sorption of Cu(II), Pb(II) and Cd(II) on MG‐Chi/Fe_3_O_4_

Metal Ion	First order			Second order		
	q_e_ (mg g^−1^)	k_1_ (min^−1^)	R^2^	q_e_ (mg g^−1^)	k_2_ (g (mg min)^−1^)	R^2^
Cu(II)	59.88	19.92	0.9748	54.94	1.31 × 10^−3^	0.9904
Pb(II)	59.17	16.11	0.9663	56.49	1.38 × 10^−3^	0.9937
Cd(II)	58.82	20.92	0.9743	53.47	1.32 × 10^−3^	0.9902

### 3.5 Reusability of MG‐Chi/Fe_3_O_4_ beads

**FIGURE 8:**
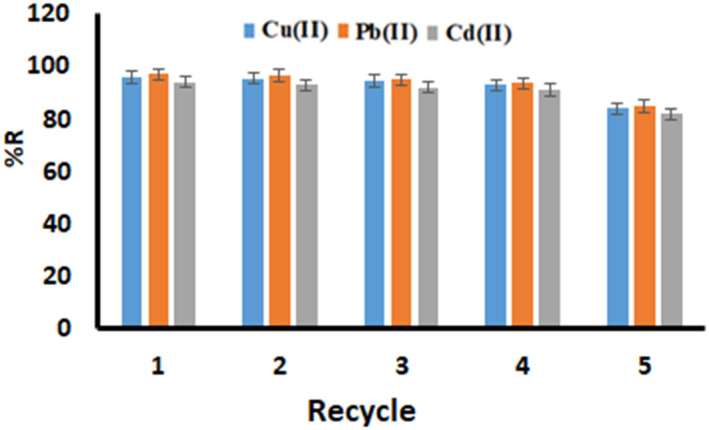
Reusability of MG‐Chi/Fe_3_O_4_ beads

The relative recoveries of the metal ions by the magnetic nanobiosorbent used in this study were determined to evaluate the reusability, and it was found that the relative recoveries had good after at least four adsorption–desorption cycles (Figure 8). The slight decrease in the relative recoveries could be due to the loss of magnetic nanobiosorbent or the disappearance of irreversible adsorption sites. Therefore, the magnetic nanobiosorbent along with proper relative recoveries of metal ions from the aqueous solution also show good reusability after the fourth elution cycle.

### 3.6 Validation of the method

The analytical performance of this method under the optimised conditions was investigated and presented in Table 3. The coefficients of determination (*r* ^2^) were 0.9993, 0.9968, and 0.9976 for Cu(II), Pb(II), and Cd(II), respectively, in the calibration curves. The limit of detection (LOD) was calculated according to the IUPAC definition, LOD = 3 SD/m, where SD is the standard deviation of 10 consecutive measurements of blank solutions and m is the slope of the calibration curves [35]. The concentration limits of detection were 0.22, 0.24, and 0.1 μg L^−1^ for Cu(II), Pb(II), and Cd(II), respectively. The limit of quantification was 0.73, 0.80, and 0.33 μg L^−1^ for Cu(II), Pb(II), and Cd(II), respectively. Linearity was maintained at 5–1000 μg L^−1^ for Cu(II) and Pb(II) and 2.5–1000 μg L^−1^ for Cd(II). The intraday and interday relative standard deviations (RSDs) were calculated. This reflects the favourable precision of the total procedure. Preconcentration factors were calculated using the ratio of analyte concentration in the solid phase (C_1_) to the initial concentration of analyte (C_0_) in the sample solution: PF = C_1_/C_0_ [36]. The extraction percentage (%E) was also calculated and with 110, 120, and 100 obtained for Cu(II), Pb(II), and Cd(II), respectively.

**TABLE 3:** Analytical figures of merit for the determination of Cu(II), Pb(II) and Cd(II) by MG‐Chi/Fe_3_O_4_ as DSPE adsorbent in water sample

Metal ion	LOD (μg L^−1^)	LOQ (μg L^−1^)	%RSD (*n* = 3)		EF	LDR (μg L^−1^)	%E	R^2^
			Intraday	Interday				
Cu(II)	0.22	0.73	1.5	2.8	55	5–1000	110	0.9993
Pb(II)	0.24	0.80	2.5	3.0	60	5–1000	120	0.9968
Cd(II)	0.10	0.33	3.0	3.5	50	2.5–1000	100	0.9976

*Note: n* = 3.

Abbreviations: DSPE, dispersive solid‐phase extraction; LOD, limit of detection; RSD, relative standard deviation; LDR, linear dynamic range.

### 3.7 Real samples analysis

In order to assess the accuracy of the developed method, samples of industrial wastewater were collected from Iran Khodro Company's wastewater (Tehran, Iran) in a clean polyethylene bottle and after filtration with membrane filter with pore size of 0.45 μm (Millipore, Merck) to eliminate the solid pollutants, under the optimum conditions (*V* = 100 ml, pH = 5.5, wt of magnetic nanobiosorbent = 20 mg, extraction time = 5 min, eluent volume = 2 ml EDTA 0.1 M, and eluent time = 2 min) and spiked wastewater samples at two concentration levels (50 μg L^−1^ and 100 μg L^−1^) of metal ions, RSDs for three replicate measurements and relative recoveries (R%) were evaluated and reported in Table 4. The relative recoveries were obtained at up to 100 with RSD (%) in the range of 1.5%–2.8%. The results in Table 3 demonstrate that the adsorbent in preconcentration and determination of Cu(II), Pb(II), and Cd(II) in a complex matrix such as industrial wastewater samples had good efficiency.

**TABLE 4:** Determination of Cu(II), Pb(II), and Cd(II) ions in Iran Khodro's wastewater sample by MG‐Chi/Fe_3_O_4_

Metal Ion	C _added_ (μg L^−1^)	C _determined_ (μg L^−1^)	RSD (*n* = 3)	%R
Cu(II)	50	50.27	1.5	100.05
	100	100.30	2.5	100.10
Pb(II)	50	50.52	2.5	100.99
	100	100.68	1.8	101.02
Cd(II)	50	51.00	2.8	102.40
	100	100.80	1.5	100.25

*Note: n* = 3.

Abbreviation: RSD, relative standard deviations.

### 3.8 Comparison of the developed method with previously reported methods

As shown in Table 5, synthesised adsorbent and the present method have better LOD, linearity, extraction time, and recovery than previously reported methods, which is an important factor in the synthesis of adsorbent for preconcentration and determination of several simultaneous metal ions [37, 38, 39, 40, 41]. Despite the modified chitosan functional groups (e.g. __COOH, __NH_2_, __CN, and especially __S^__^), the formation of dative and coordination bonds between the metal ions and the adsorbent is the main driving force for the adsorption process [24]. Therefore, this method was applied for the successful simultaneous determination of Cu(II), Pb(II), and Cd(II) levels in industrial wastewater samples.

**TABLE 5:** Comparison of the proposed method with some of the methods reported in the literature for determination of the metal ions

Metal Ion	Adsorbent	LOD (μg/L^−1^)	LDR (μg/L^−1^)	Extraction time (s)	%Recovery	Ref.
Cu(II)	Core−shell Fe_3_O_4_ polydopamine nanoparticles	0.22	15–750	5	90–99	[37]
Cd(II)	Magnetic chitosan hydrogels	0.20	0.5–250	120	96.0–110.4	[38]
Pb(II)	Modified multiwalled carbonnanotubes	0.26	2.0–25.0	600	97	[39]
Cu(II)	MWCNT‐Bi_2_S_3_ nanomaterial	3.98	^a^	120	92–100	[40]
Pb(II) Cd(II) Cu(II)	Magnetic allylamine Modified graphene oxide‐poly (vinyl acetate‐co‐divinylbenzene) Nanocomposite	2.390.372.34	^a^	300	96–102	[41]
Cu(II) Pb(II) Cd(II)	Chi‐MG/Fe_3_O_4_	0.220.240.10	5–10005–10002.5–1000	300	100–102	This study

^a^ Not reported.

## 4 CONCLUSIONS

In this study, a magnetic nanobiosorbent was synthesised in order for preconcentration and determination of Cd(II), Cu(II), and Pb(II) levels from wastewater samples. “During synthesis, several functional groups, including __NH2, __CN__ and __S__ groups, are formed on the surface of magnetic nanobiosunts that can adsorb the metal ions.” The good preconcentration factor and the significant extraction percentage for analysis of real samples showed the efficiency of the proposed method. The magnetic nanobiosorbent can be easily separated from the complex matrix by a magnet, enabling a rapid adsorption process. The developed method was optimised and validated, and showed low LODs, good linearity, and good relative recoveries, with less consumption of organic solvent and sorbent for the simultaneous determination of Cd(II), Cu(II), and Pb(II) in wastewater samples.

## CONFLICT OF INTEREST

The authors have declared that they have no conflict of interest.
